# Clinical, Immunological, and Neuroimaging Characteristics and Outcome Profile of Neurocysticercosis in Children at a Neurology Department in Antananarivo: A Retrospective Observational Study

**DOI:** 10.7759/cureus.114048

**Published:** 2026-08-06

**Authors:** Julien Razafimahefa, Rivo Lova Herilanto Rakotomalala, Odilon Rahamefy, Lala A Rajaonarison, Anjanirina Rahantamalala, Alain D Tehindrazanarivelo

**Affiliations:** 1 Neurology, Joseph Raseta Befelatanana University Hospital, Antananarivo, MDG; 2 Pediatrics, Tsaralalana Mother and Child University Hospital, Antananarivo, MDG; 3 Neurology, Professeur Zafisaona Gabriel (PZAGA) University Hospital, Mahajanga, MDG; 4 Immunology, Pasteur Institute Madagascar, Antananarivo, MDG

**Keywords:** clinical, clinical and radiological, developping country, epilepsy, immunological, madagascar, neurocysticercosis, neuroimaging findings, outcomes, pediatric

## Abstract

Introduction: Madagascar is an endemic area for neurocysticercosis (NCC). This study aimed to describe the clinical, immunological, and neuroimaging findings, as well as the clinical and radiological outcomes, in Malagasy children.

Methods: This retrospective, descriptive, observational cohort study was conducted from October 2013 to October 2017 in the neurology department of a hospital in Antananarivo. Pediatric cases of NCC were diagnosed using brain CT and serological tests (serum/cerebrospinal fluid). Clinical follow-up was performed for all patients, while CT/MRI follow-up was carried out only in active cases.

Results: Of the 125 patients enrolled, 30 (24%) were included in the study. The cohort consisted of 16 boys and 14 girls, with a mean age of 9.6 years (range: 2-15 years). The main symptoms were epilepsy in 26 patients (86.6%), headaches in three (10%), and dystonia in one (3.3%). Imaging revealed isolated parenchymal lesions in 11 cases (36.6%), isolated meningeal involvement in six cases (20%), and mixed involvement (parenchymal and meningeal) in 13 cases (43.3%). Analysis of outcomes showed stable epilepsy in cases with isolated parenchymal lesions or solitary calcified lesions, whereas seizure recurrence was observed in cases with mixed or multiple lesions.

Discussion: A high frequency of parenchymal and multiple meningeal forms was observed in Malagasy children. Early infection appears to be common, preceding the typical 5-10-year latency period required for lesion calcification. The long-term prognosis is poor in mixed forms, with a risk of drug-resistant epilepsy.

Conclusion: Pediatric NCC requires an innovative approach to diagnosis and therapeutic management.

## Introduction

Neurocysticercosis (NCC), a neglected tropical disease, is estimated to affect 2.5 to 3.8 million people living with epilepsy, according to the World Health Organization. NCC is the most frequent cause of epileptic seizures in children in developing countries, particularly in Latin America, Asia, and sub-Saharan Africa [1]. Its prevalence varies from 20% to 30%, depending on the country and the methodology used in studies, such as general population-based or hospital-based studies. A seroprevalence study of cysticercosis using antigen ELISA (enzyme-linked immunosorbent assay) serology in school-aged children in the general population of Madagascar reported hyperendemicity [2]. Previous Malagasy studies on NCC in children were conducted in hospital pediatric departments as case series, utilizing increasingly advanced diagnostic tools, including immunological tests and brain CT scans, but made no specific mention of identifying a scolex during neuroimaging interpretation [3,4]. A Mexican study reported differences in clinical presentation, radiological findings, and inflammatory status between NCC in adults and children [5].

This study aims to describe the main clinical, immunological, neuroimaging, and evolutionary (clinical and radiological) characteristics of NCC in Malagasy children followed in the Adult Neurology Department of Befelatanana University Hospital (CHU).

## Materials and methods

This was an observational, retrospective, descriptive cohort study conducted among patients who attended the Neurology Department of the Joseph Raseta Befelatanana University Hospital Center in Antananarivo, Madagascar, between October 2013 and October 2017. This department provides conventional inpatient care for adults with neurological disorders, as well as outpatient consultations and day-hospital services for patients of all ages, including children. A neurocysticercosis registry was established in 2005 in collaboration with the Pasteur Institute of Madagascar. Children included in the present study were subsequently identified from this registry.

The eligibility criteria included all children under 15 years of age who attended consultations at the neurology department during the study period for new-onset epileptic seizures or progressive headaches. They were included if they could undergo neuroimaging, such as a brain CT scan or MRI, according to their parents' financial ability. If neuroimaging revealed active lesions but did not identify a scolex, immunological testing was performed to fulfill the international diagnostic criteria for neurocysticercosis proposed by Del Brutto et al. [6]. Patients who could not undergo neuroimaging were not included, and patients with diagnoses other than neurocysticercosis, based on clinical symptoms and neuroimaging results, were excluded.

This was a retrospective study based on data already collected at the neurology department. Patients verbally consented to receive treatment as part of the routine management of their conditions at the university hospital. Data were anonymized, and confidentiality was maintained. Human rights were respected.

Sociodemographic, clinical, and follow-up data were collected from the neurocysticercosis registry and patients' medical records. Variables included age, sex, medical symptoms, initial clinical manifestations, and symptoms observed during follow-up, particularly epileptic seizures, headaches, and any secondary cognitive impairment.

All brain imaging examinations performed at diagnosis, including computed tomography (CT) scans and/or magnetic resonance imaging (MRI), were reviewed by the medical team to confirm the diagnosis of neurocysticercosis and exclude alternative diagnoses. The choice between a brain CT scan and MRI was guided first by financial constraints because many patients could not afford MRI and paid for CT scans themselves, and second, if definitive neuroimaging findings, such as a scolex or calcifications, were seen on a brain CT scan, MRI was not required. Lesions were subsequently classified according to their location (parenchymal or meningeal forms), stage of evolution (active or inactive/calcified lesions), and number.

Clinical follow-up was assessed using data recorded during follow-up consultations in the patients' medical records. The evolution of neurological manifestations, including the persistence, recurrence, or resolution of epileptic seizures and headaches, as well as the occurrence of neurological or cognitive complications, was analyzed.

Patients presenting with active neurocysticercosis lesions on neuroimaging received antiparasitic treatment with albendazole at a dose of 20 mg/kg/day for eight days. This treatment was combined with prednisone at a dose of 1 mg/kg/day for five days to reduce the inflammatory reaction associated with parasite destruction. In cases of significant cerebral edema or severe intracranial hypertension, prolonged corticosteroid therapy could be prescribed according to the patient's clinical and radiological evolution. A follow-up brain CT scan was performed three to six months after treatment to assess lesion progression.

Patients with epileptic seizures received antiepileptic therapy tailored to the seizure type and clinical course, according to the protocols used in the department.

Data entry and statistical analyses were performed using Microsoft Excel (Microsoft Corporation, Redmond, Washington) and Epi Info 7 software (Centers for Disease Control and Prevention, Atlanta, Georgia).

Associations between categorical variables were assessed using the chi-square test when expected frequencies were ≥5, whereas Fisher's exact test was used when expected frequencies were <5. Statistical significance was set at p < 0.05.

## Results

Study population

There were 125 children under 15 years of age who attended consultations at the neurology department during the study period for new-onset epileptic seizures or progressive headaches. Among them, 54 children who were able to undergo neuroimaging, such as a brain CT scan or MRI, were included. Seventy-one children who could not undergo neuroimaging were not included, and 24 children had diagnoses other than neurocysticercosis based on clinical symptoms and neuroimaging results and were excluded. The final study population consisted of 30 children.

Sociodemographic characteristics

The mean age of the study population was 9.6 years (range: 2-15 years). The male-to-female ratio was 1.1, with 16 boys and 14 girls. Most children (96%) resided in Antananarivo, while the remaining patients came from other provinces.

Main clinical manifestations

Epileptic seizures were the main clinical manifestation in 26 patients (86.6%), with or without associated learning difficulties. A focal epileptic onset was observed in 85% of the children with epilepsy. Isolated headaches were reported in three patients (10%). One child (3.3%) presented with dystonic movements. Three patients (10%) had learning difficulties associated with their epileptic seizures.

Neuroimaging findings

Brain CT was the most accessible neuroimaging modality for the majority of patients. Two patients underwent brain MRI.

Having received larvicidal treatment before neurological follow-up, one patient developed diffuse encephalitis with marked cerebral edema, recurrent epileptic seizures, cognitive deterioration, and diffuse cerebral atrophy (widening of the cortical sulci and ventricular dilatation) at the end of the inflammatory process (Figure 2).

Lesions suggestive of NCC were predominantly of mixed localization (parenchymal and meningeal) in 13 cases (43.3%), isolated parenchymal localization in 11 cases (36.6%), and isolated meningeal localization in six cases (20%). Among the six meningeal cases, one child presented with hydrocephalus, and five had calcified nodules.

Multiple lesions were the most common finding (15 cases, 50%), whereas single lesions were observed in 13 cases (46.6%). Regarding the stage of evolution, active lesions predominated and were identified in 23 children, while calcified lesions were found in seven cases (23.3%) (Table 1). 

**Table 1:** Neuroimaging characteristics of neurocysticercosis lesions in children in the Neurology Department at Befelatanana University Hospital *Non-cystic lesion: hydrocephalus (not counted as a lesion). **Active: parenchymal ring-enhancing lesion with or without a scolex, nodular lesion with peripheral edema, or active hydrocephalus. ***Healing: complete resolution or scarring with calcification.

Topography	Lesion number (N = 29)			Lesion type (N = 30)		Follow-up brain CT scan (N = 30)		
	Unique, n(%)	Multiple, n(%)	Non-cystic lesion*, n(%)	Active**, n(%)	Calcification, n(%)	Healing***, n(%)	Many stages, n(%)	Calcification at first CT scan follow-up not done, n(%)
Meningal	3 (50.3)	2 (33.6)	1 (16.6)	1 (13.6)	5 (83)	1 (16.6)		5 (83.3)
Parenchymal	10 (90.9)	1 (9.09)		10 (90.9)	1 (9.09)	11 (100)		
Mixt	1 (7.6)	12 (92.3)		12 (92,3)	1 (7.6)	7 (53.8)	6 (46.15)	
Total	14 (46.6)	15 (50)	1 (3.3)	23 (76.7)	7 (23.3)	19 (63,33)	6 (20)	5 (16.6)

Brain MRI in two children showed a single active parenchymal lesion presenting as a ring-enhancing cystic lesion with a scolex inside, surrounded by peripheral edema, particularly on T1-weighted images with gadolinium injection. Most parenchymal lesions exhibited ring enhancement. A scolex was identified in approximately one-third of the cases (Table 2). 

**Table 2:** Children neurocysticercosis diagnostic criteria according to Del Brutto [6] EITB: enzyme-linked immunoelectrotransfer blot, ELISA: enzyme-linked immunosorbent assay.

Case	Clinic	Neuroimaging						Serology ELISA/EITB blood CSF
	Symptoms	Topography	Ring enhance	Visible scolex	Active lesion	Characteristic calcification	Follow-up CT scan	
1	Epilepsy	Meningeal	N/A	N/A	No	Yes	N/A	N/A
2	Epilepsy	Meningeal	N/A	N/A	No	Yes	N/A	N/A
3	Headache	Meningeal	N/A	N/A	Yes	No	Healing	Positive
4	Epilepsy	Mixt	Yes	No	No	Yes	atrophy	Positive
5	Dystonia	Parenchymal	N/A	Yes	Yes	No	calcification	Positive
6	Epilepsy	Meningeal	N/A	N/A	No	Yes	N/A	N/A
7	Epilepsy	Meningeal	N/A	N/A	No	Yes	N/A	N/A
8	Epilepsy	Meningeal	N/A	N/A	No	Yes	N/A	N/A
9	Epilepsy	Parenchymal	Yes	Yes	Yes	No	Calcification	N/A
10	Epilepsy	Parenchymal	Yes	Yes	Yes	No	Calcification	Negative
11	Epilepsy	Mixt	Yes	Yes	Yes	Yes	Calcification	Positive
12	Epilepsy	Mixt	Yes	No	Yes	Yes	Calcification	Positive
13	Epilepsy	Parenchymal	Yes	No	Yes	No	Healing	Positive
14	Epilepsy	Parenchymal	Yes	No	Yes	No	Calcification	Positive
15	Epilepsy	Parenchymal	Yes	Yes	Yes	No	Healing	Positive
16	Epilepsy	Parenchymal	Yes	Yes	Yes	No	Calcification	Negative
17	Epilepsy	Parenchymal	Yes	Yes	Yes	No	Healing	Positive
18	Headache	Mixt	Yes	No	Yes	Yes	Calcification	Positive
19	Epilepsy	Parenchymal	Yes	Yes	Yes	No	Healing	Negative
20	Epilepsy	Parenchymal	Yes	Yes	No	Yes	Healing	N/A
21	Epilepsy	Mixt	Yes	No	Yes	No	Calcification	Positive
22	Epilepsy	Mixt	N/A	N/A	Yes	Yes	Calcification	N/A
23	Epilepsy	Parenchymal	N/A	N/A	Yes	No	Healing	Positive
24	Epilepsy	Mixt	N/A	N/A	Yes	No	Calcification	N/A
25	Epilepsy	Mixt	Yes	No	Yes	Yes	Calcification	Negative
26	Headache	Mixt	Yes	No	Yes	Yes	Calcification	Positive
27	Epilepsy	Mixt	Yes	No	Yes	Yes	Calcification	Negative
28	Epilepsy	Mixt	Yes	No	Yes	Yes	Calcification	Positive
29	Epilepsy	Mixt	Yes	No	Yes	Yes	Calcification	Positive
30	Epilepsy	Mixt	Yes	No	Yes	Yes	Calcification	Negative

NCC serology in blood or cerebrospinal fluid

Among the 21 patients who underwent serological testing, including those with active lesions and those in whom only blood sampling was performed when lumbar puncture was not feasible because of parental or patient (children) refusal, 15 cases (71.4%) had positive ELISA and EITB (enzyme-linked immunoelectrotransfer blot) serology. These patients were among those presenting with mixed or parenchymal lesions (χ² = 10.4; p = 0.003).

Clinical outcomes

The course of epilepsy remained stable in 18 patients (60%). Recurrent epileptic seizures were recorded in 10 patients (33.3%). These recurrences were more frequent among patients with multiple lesions (Fisher's exact test, p = 0.001).

Seven patients (23.3%) developed cognitive impairment, mainly learning difficulties, during follow-up. These patients had multiple NCC lesions and recurrent epileptic seizures (Fisher's exact test, p = 0.01). Two patients did not experience epilepsy but presented with movement disorders and isolated headaches.

Follow-up brain CT findings

Children with active lesions on initial brain imaging underwent follow-up CT scans three or six months after specific treatment. Overall, 19 of the 23 children (82.6%) achieved radiological cure, defined as complete disappearance of the lesions or residual calcifications without any evidence of active NCC.

One child with hydrocephalus recovered after medical treatment; however, follow-up CT revealed residual cortico-subcortical atrophy.

**Figure 1:**
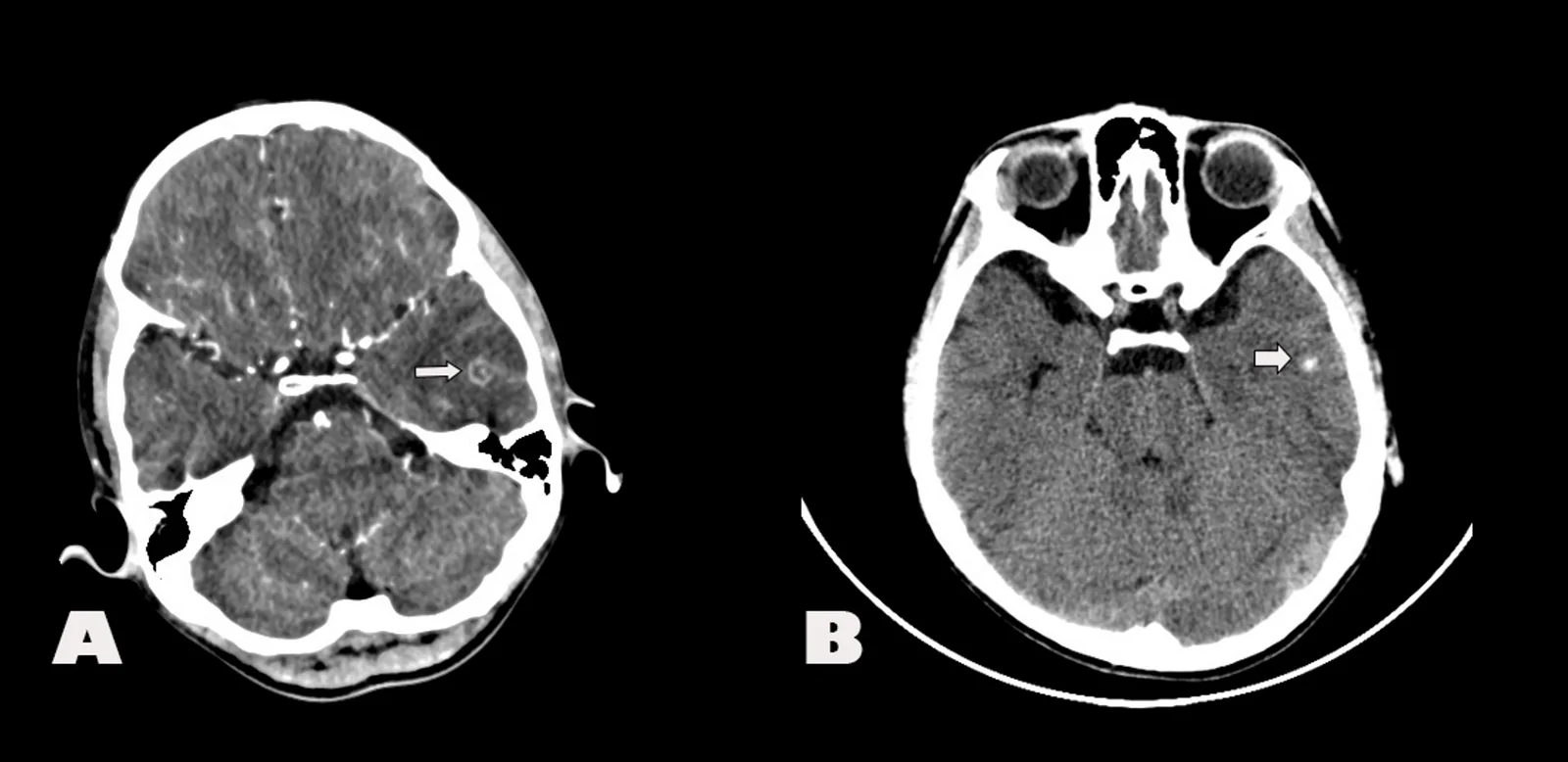
Brain CT scan of a nine-year-old boy showing a ring-enhancing cyst with a scolex inside, located in the temporal lobe (A), and scarring with calcification three months after albendazole treatment (B)

In five children, calcification of previously active lesions was documented. However, follow-up CT scans performed after recurrent epileptic seizures revealed several newly active lesions, followed by a tendency toward diffuse cerebral atrophy with widening of the cortical sulci and ventricular dilatation. Among these five children with ongoing inflammatory activity, a nine-year-old child presented with multiple active lesions (Figure 1). 

**Figure 2:**
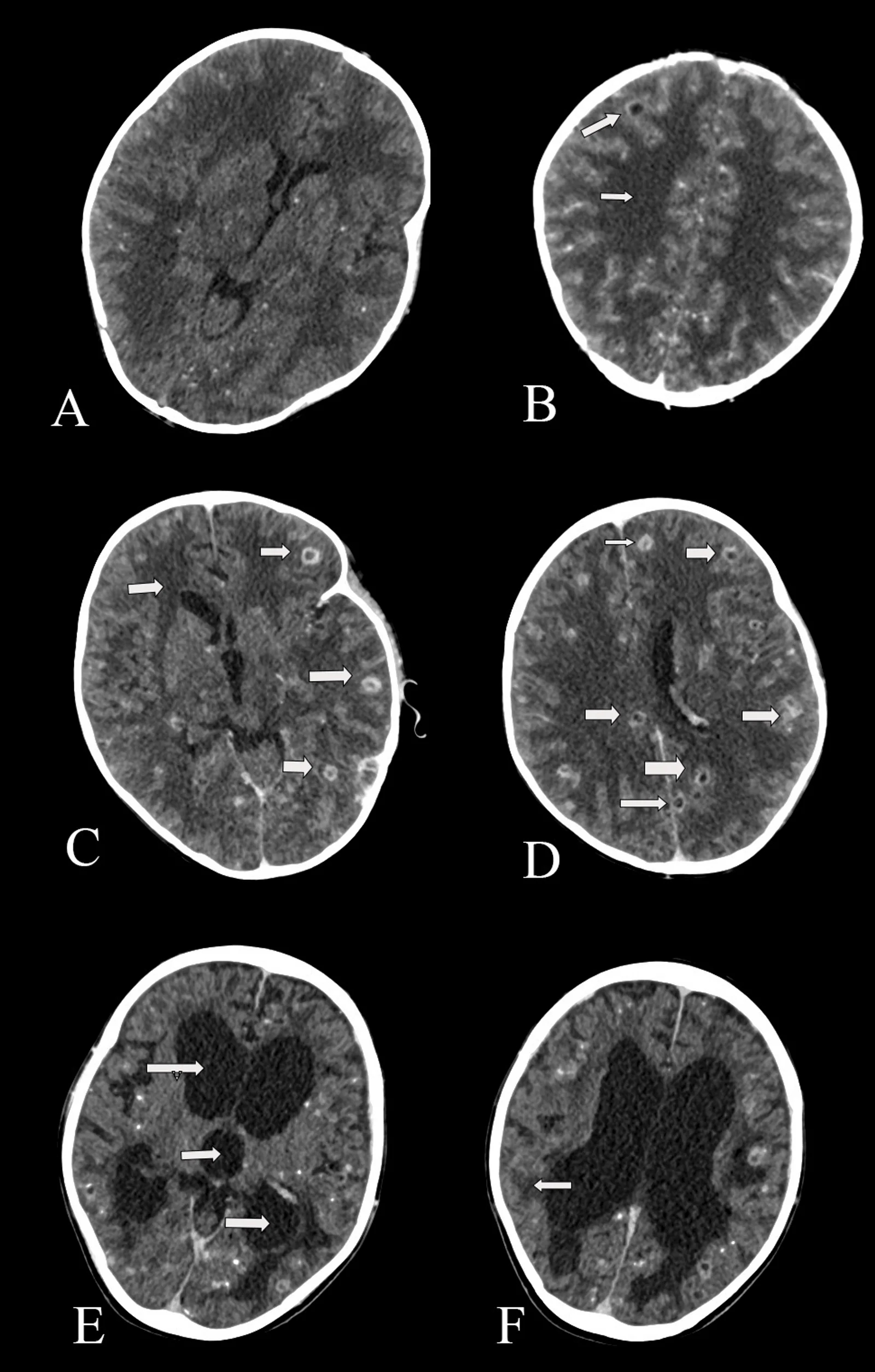
Brain CT scan of a seven-year-old boy with massive ring-enhancing neurocysticercosis lesions and extensive edema after albendazole treatment (A, B); additional ring-enhancing lesions appearing six months later (C, D); and the final evolutionary stage with calcifications, cortical and subcortical atrophy, and ventricular enlargement (E, F)

Having received larvicidal treatment before neurological follow-up, this child subsequently developed diffuse encephalitis with marked cerebral edema, recurrent epileptic seizures, cognitive deterioration, and diffuse cerebral atrophy (widening of the cortical sulci and ventricular dilatation) at the end of the inflammatory process (Figure 2).

Diagnostic confirmation according to Del Brutto et al. [6]

All patients fulfilled the diagnostic criteria for NCC based on the following findings: identification of a scolex within a ring-enhancing lesion (absolute criterion); positive ELISA/EITB immunological results in blood or cerebrospinal fluid, together with complete resolution or calcification of lesions on follow-up brain CT scans for highly suggestive lesions, particularly in cases in which no scolex was identified within cystic lesions or in cases of hydrocephalus without a compressive mass, corresponding to major neuroimaging and exposure criteria, associated with minor suggestive clinical manifestations and the Malagasy epidemiological context (Table 2).

## Discussion

This study highlights the progress achieved in the diagnostic and therapeutic management of NCC in children among cases managed in the adult neurology department in Antananarivo.

Several methodological differences distinguish this study from the previous two Malagasy reports [3,4]. Children with NCC were recruited from the adult neurology department of Befelatanana University Hospital. This adult neurology department is part of the national referral hospital and manages adult inpatients and outpatients of all ages at the consultation and day-hospital units for diagnosis, specialized management, and neurological consultation. Madagascar does not yet have a pediatric neurologist or a pediatric neurology department. Although this approach may have introduced recruitment bias, it reflects the role of the department's outpatient and day-hospital units, which manage children with neurological disorders in collaboration with the pediatric department. A Mexican research group investigating pediatric NCC [5] similarly recruited children from neurology and neurosurgery departments.

Brain imaging (CT or MRI) was used as the first-line diagnostic investigation to identify a scolex (an absolute diagnostic criterion) or lesions highly suggestive of NCC while excluding alternative causes of neurological manifestations in children [6]. When imaging findings suggested NCC without definitive diagnostic features, ELISA and EITB serological tests (performed on serum or CSF) were used to support the diagnosis [7]. In the present study, when CSF examination was not feasible, only serum serology was performed, and follow-up CT findings ultimately contributed to diagnostic confirmation.

Follow-up brain CT scans were performed three or six months after antiparasitic treatment for active forms of NCC. This interval has been widely validated for assessing the efficacy of anthelmintic therapy [8,9]. Complete disappearance or healing of a lesion suspected of NCC after antiparasitic treatment constitutes a major neuroimaging criterion according to current recommendations.

In this pediatric series, the clinical presentation of NCC was predominantly characterized by focal-onset epileptic seizures. NCC is one of the leading causes of epilepsy in developing countries [10]. In endemic regions, as elsewhere in the world, NCC frequently presents with seizures regardless of age [7,10]. The prevalence of NCC in the pediatric population is often extrapolated from epilepsy prevalence estimates [5,11]. Headaches related to intracranial hypertension commonly occurred following seizures. These headaches are associated with inflammatory and edematous reactions surrounding parenchymal lesions, as well as active meningeal forms such as hydrocephalus or racemose NCC [12].

Access to neuroimaging has substantially improved in Madagascar owing to the increasing availability of CT facilities in provincial areas and the introduction of MRI in radiology centers in Antananarivo. However, access remains dependent on public health coverage, private health insurance availability, and patients' financial resources. In the present study, review of imaging records allowed identification of a scolex within ring-enhancing lesions, thereby improving diagnostic accuracy. Complete resolution or calcification of ring-enhancing lesions without a visible scolex on follow-up CT also supports the diagnosis of NCC, regardless of serological status [6]. Meningeal NCC in this study was characterized by meningeal calcifications and one case of hydrocephalus with positive CSF serology that resolved after anthelmintic treatment. No racemose lesions were identified. Indeed, racemose NCC is reported to be more common in adults than in children according to a comparative Mexican study [5].

ELISA and EITB are indirect immunological diagnostic methods for NCC that detect antibodies against Taenia solium. A positive blood test indicates exposure to the parasite, whereas positivity in CSF is considered strong evidence of active NCC [6]. These tests are available at the Pasteur Institute of Madagascar. In this study, children with ring-enhancing parenchymal lesions without a visible scolex and without active meningeal disease underwent serological testing. Blood testing was more readily accepted by parents and children than lumbar puncture. Positive results in blood or CSF provided additional evidence supporting the diagnosis when associated with complete resolution or calcification of active lesions on follow-up CT after anthelmintic therapy.

From a clinical perspective, epilepsy remained stable in the majority of children. Most seizure-free patients presented with a single parenchymal lesion. In contrast, patients with mixed localization and multiple lesions experienced more frequent seizure recurrence. An Indian study reported that children with a single lesion have a more favorable seizure outcome, whereas multiple lesions are associated with a higher risk of recurrence and poorer seizure control [13].

The youngest child in this cohort was approximately two years old and presented with epileptic seizures associated with a calcified lesion on brain imaging. Calcification of a cystic lesion generally occurs within 5 to 10 years, depending on the host's inflammatory and immune response [14]. This finding suggests very early infestation, potentially related to maternal hygiene practices in endemic areas. All children included in this study initially had normal psychomotor development. Learning difficulties and cognitive slowing were observed in seven children with recurrent seizures and multiple lesions on brain CT. Similar findings have been reported in an Indian study [15]. In our series, the most severe cases were associated with repeated inflammatory reactions caused by progressive degeneration of multiple lesions, potentially leading to encephalitis and ultimately residual cerebral atrophy. Massive cerebral NCC in children and young women is considered a high-risk form because of the intense inflammatory response triggered by multiple parasitic lesions [16].

In this study, active forms of NCC were treated with albendazole at a dose of 20 mg/kg/day for eight days, combined with corticosteroid therapy (prednisone 1 mg/kg/day for five days). Follow-up CT scans performed three or six months later confirmed healing of active lesions through either complete resolution or calcification, particularly in cases with single lesions. The efficacy and good tolerance of albendazole in promoting lesion resolution have been previously demonstrated [17]. In children with multiple lesions, active lesions evolved toward calcification; however, this process was accompanied by inflammatory reactivation of other degenerating lesions, requiring renewed corticosteroid therapy and a second course of albendazole. Panday et al. reported that three cycles of albendazole treatment in patients with multiple lesions resulted in complete lesion disappearance [18].

Combination therapy with albendazole and praziquantel has been shown to be more effective than albendazole alone in non-encephalitic forms of NCC. However, in the present study, the risk of severe inflammatory reactions in patients with multiple lesions limited the use of combined anthelmintic therapy. Even under albendazole monotherapy, one child with multiple lesions developed severe encephalitis. This child, who developed encephalitis after initiation of albendazole treatment before neurological management, had not received concomitant corticosteroid therapy. Management of the encephalitic episode required higher-dose corticosteroids (2 mg/kg/day), followed by gradual tapering to achieve complete resolution of inflammation. Corticosteroid therapy remains the cornerstone of treatment for encephalitic NCC, whereas combined anthelmintic therapy is considered potentially hazardous because of the risk of exacerbating cerebral inflammatory reactions [19].

## Conclusions

This study highlights the importance of neurocysticercosis as a cause of epilepsy in children in endemic areas such as Madagascar. Accessibility to neuroimaging has improved, although it remains frequently limited to hospital patients. Identification of a scolex on neuroimaging strengthens the diagnosis of parenchymal neurocysticercosis and should be an important step during neuroimaging interpretation. Immunological testing available in Madagascar could help improve diagnostic tools. The results suggest that both single parenchymal lesions and multiple lesions should be considered on neuroimaging in Malagasy children and that children with a single lesion may have better epilepsy control than those with multiple active lesions. Further multicenter prospective studies should be the next step toward developing national recommendations for the management of neurocysticercosis in children.
